# Prevalence and determinants of pulmonary hypertension in rheumatic heart disease patients at university of Gondar comprehensive specialized hospital: a retrospective study from 2018 to 2023

**DOI:** 10.1186/s12872-025-04648-1

**Published:** 2025-03-18

**Authors:** Fikadu Alemiye Molla, Desalew Getahun Ayalew, Hailemaryam Alemu Astatk, Abebe worku Teshager, Gebrehiwot Lema Legesse, Daniel Belay Agonafir, Shibabaw Fentahun, Belete Sisay Assefa, Tilahun Nega Godada, Deresse Abebe Gebrehana, Abilo Tadesse

**Affiliations:** 1 Department of Internal Medicine, School of Medicine, College of Medicine and Health Sciences, University of Gondar, P. O. Box 196, Gondar, Ethiopia; 2 Department of Internal Medicine, School of Medicine, College of Medicine and Health Sciences, Wachemo University, Hossana, Ethiopia; 3 Department of Internal medicine, Menelik ll Comprehensive specialized Hospital, Addis Ababa, Ethiopia

**Keywords:** Rheumatic heart disease, Valvular heart disease, Pulmonary hypertension, Factors, Prevalence, Ethiopia

## Abstract

**Introduction:**

Most Rheumatic heart disease patients present with advanced disease and complications, pulmonary hypertension being one of the main complications. The presence of pulmonary hypertension is independently associated with increased pre-operative and perioperative morbidity and mortality in RHD patients. There are only few studies that showed the magnitude of pulmonary hypertension in RHD patients. This study was done to know the magnitude and predictors of PH in RHD patients so that early identification and intervention can be done for those at risk to develop PH.

**Methods:**

An institution-based retrospective study was conducted by reviewing medical records of patients at adult echocardiographic unit from September, 2018 to September, 2023. Systolic PAP > 35mmHg using 2D echocardiography was used to diagnose pulmonary hypertension. Bi-variable logistic regression analysis followed by multivariable logistic regression analysis was done using SPSS statistics 25. P value < 0.05 and 95% CI was used to determine significant association.

**Result:**

A total of 230 RHD patients were included during the study period between September 2018 and September 2023. Most patients were below the age of 35 years (68.3%) with median age of 28 years and interquartile range of 21 to 38 years. Among the study participants 72.2% were females and 67% of them were rural residents. PH prevalence among RHD patients was found to be 77.4% (95% CI: 71.4%, 82.6%). Most patients (51.3%) had severe PH (≥ 60mmHg), whereas Mild PH (36–44mmHg) was 7.4% and moderate PH (45-59mmHg) was 18.7%. PH was found to have significant association with severe MS (AOR = 5.31, 95%CI: 1.87, 15.06), moderate to severe MR (AOR = 2.68, 95% CI: 1.05, 6.84), NYHA functional class III and IV (AOR = 2.60, 95% CI: 1.01, 6.68) and Diuretics use (AOR = 4.43, 95% CI: 1.33, 14.70).

**Conclusion:**

The prevalence of PH among rheumatic heart disease patients in this study was high. Moderate to severe MR, severe MS, NYHA class III and IV, and diuretics use were significantly associated with PH. Expanding surgical intervention to address this patients is needed to decrease PH prevalence and morbidity and mortality associated with it.

**Supplementary Information:**

The online version contains supplementary material available at 10.1186/s12872-025-04648-1.

## Introduction

According to estimates from 2015, Rheumatic fever and Rheumatic Heart Disease (RHD) affects around 33.4 million people globally, resulting in 319,400 deaths due to RHD [1]. RHD is a major non-communicable disease in low and middle-income countries, particularly in sub-Saharan Africa. It occurs following a bacterial infection called Group A streptococcal (GAS), which leads to acute rheumatic fever (ARF) and can ultimately cause RHD [2, 3].

It has been observed that approximately 50% of patients who are newly diagnosed with rheumatic heart disease already have advanced disease and complications. The most commonly observed complications are heart failure and pulmonary arterial hypertension [2]. The majority of patients have moderate to severe valvular lesions during diagnosis, which require surgical interventions. However, the use of percutaneous and surgical interventions is significantly low in low-income countries compared to high and middle-income countries, despite the fact that a greater number of patients with rheumatic heart disease require these interventions in low-income countries [4, 5].

Pulmonary hypertension (PH) is a common complication of cardiac diseases and is often a sign of advanced disease, especially in cases of valvular heart disease. It is independently associated with increased perioperative morbidity and mortality. Studies have shown that Pulmonary hypertension in RHD is linked to decreased functional capacity, increased perioperative and long-term mortality, as well as post-operative residual PH and right ventricular dysfunction [6, 7].

The prevalence of pulmonary hypertension (PH) in RHD (rheumatic heart disease) is not clearly defined due to the various diagnostic methods used in different studies. Different centers have reported a wide range of prevalence, ranging from 11 to 72.1%. However, a large prospective cross-sectional study that involved 3343 patients from 25 hospitals across Africa, India and Yemen reported that the prevalence of PH in RHD patients was 28.8% [2, 4, 8–10]. There have been only a few studies that have attempted to identify factors associated with PH in RHD patients. Many factors contribute to the development of PH in heart failure patients, including the patient’s age, severity of valvular lesions, size and volume of the left atrium (LA), left ventricular end-diastolic (LVED) diameter, non-cardiac comorbidities, as well as the duration and degree of symptoms [11–14]. 

Various methods are used to diagnose pulmonary hypertension in different studies. While most hospital-based studies rely on Trans-thoracic echocardiography for diagnosing PH, the gold standard method is right heart catheterization. However, this method is invasive and not easily accessible in many centers, especially in developing countries such as Ethiopia. In cases of rheumatic heart disease patients, Trans-thoracic echocardiography can accurately measure the systolic pulmonary arterial pressure (SPAP) with acceptable accuracy [6].

Patients diagnosed with RHD require timely surgical intervention to prevent a range of complications including advanced Heart Failure, PH, and thromboembolic events. However, access to valvular surgery is limited in Ethiopia, with only a few hospitals and surgeons equipped to undertake such procedures. Consequently, patients in need of surgical intervention may experience extensive waiting periods, often lasting several years [15].

This study aimed to determine the prevalence of pulmonary hypertension and its associated factors among RHD patients at the echocardiography unit of the University of Gondar Comprehensive Specialized Hospital in Northwest Ethiopia.

## Methods

### Study setting, design, and population

Hospital-based retrospective cross-sectional study was conducted by reviewing the medical records of patients diagnosed with RHD in the adult echocardiography unit at the University of Gondar Comprehensive Specialized Hospital from September 2018 to September 2023. The hospital is located at Gondar town, which is 727 km far from the capital city of Ethiopia, Addis Ababa. It has many Specialties including surgery, gynecology and obstetrics, internal medicine, pediatrics, ophthalmology, radiology, dermatology, pathology, and psychiatry. It serves as catchment area of nearly 13 million people. In 5 years’ time more than 3000 trans-thoracic echocardiography was done by a cardiologist, among which there about 500 are RHD patients but there is no surgical intervention setup for RHD patients.

All patients diagnosed to have rheumatic heart disease in adult echocardiography unit of UOGCSH were Source population and all patients diagnosed with RHD during the study period were Study population, from September, 2018 to September, 2023.

### Inclusion and exclusion criteria

All patients from adult echocardiography unit of UOGCSH with the diagnosis of RHD were included whereas RHD patients with lost medical and echocardiographic record and other known causes of pulmonary hypertension were excluded.

### Sample size determination

All RHD patients during the study period were included in the study.

### Study variables

#### Dependent variable

pulmonary hypertension.

**Independent variables**:

- **Sociology-demographic factors**: Age, sex and residence.
- **Clinical characteristics and laboratory variables**: NYHA functional class, duration of symptoms, symptoms, frequency of admission, BP, Thromboembolic Events, HIV, Hemoglobin, Creatinine, serum sodium, albumin.

- **Echocardiographic variables**: Type and severity of valvular lesion, LVEDD, LVESDD, EF, LA size, RA size, RV size.

- **ECG parameters**: AF, RBBB, RVH.
- **Medications and intervention**: Diuretics, B-blocker, ACEI, Digoxin, Anticoagulant, Secondary prophylaxis, Intervention.

### Operational definition

- RHD = diagnosed based on the World Heart Federation criteria for echocardiographic.

Diagnosis of rheumatic heart disease.

- Pulmonary hypertension = estimated PASP > 35mmHg by echocardiography [16].
- PH severity - mild PH(35mmHg − 44mmHg), moderate PH (45mmHg -59mmHg), severe PH (≥ 60mmHg) [16].

- Severe MS = MVA < 1.5 cm²(ACC/AHA 2020, valvular heart disease) [17].
- Echocardiographic measurements (ASE, chamber quantification,2018 ) [18].
- EF = Preserved EF > 50%.

Reduced EF ≤ 50.

- LVEDD(mm) = 42-58for Male, 38–52 for female.

- LVESD(mm) = 25–40 for male, 22–35 for female.

- LA (mm) = 30–40 for male,27–38 for female.
- RA(ml/m²) = 21 ± 6 for male, 25 ± 7 for female.

### Data collection and materials

Data was collected from study population using data abstraction tool through detail chart review. A structured questionnaire in English was developed specifically for this study after a thorough review of relevant literature. The questionnaire is provided in the supplementary materials. The questionnaires were subdivided into four parts; Socio-demographic characteristics, clinical and laboratory, echocardiographic and ECG and medications and interventions. The latest echocardiography report done by cardiologist was used for diagnosis of PH and defining echocardiographic variables. Two general practitioners were recruited for data collection. The data collectors had been given orientations about data collection, purpose of the study and exclusion criteria.

### Data quality assurance

Structured data abstraction tool was used and the questionnaire was per-tested before the actual study had begun. The data collectors were physicians for better understanding and interpretation of patient’s medical records and data collectors were trained and closely supervised during data collection and data entry. During data collection it was checked daily for accuracy and completeness by the supervisor and principal investigator.

### Data analysis

The collected data was checked for completeness, and then entered into Epidata version 4.6 and analyzed by SPSS statistics 25. Descriptive measures such as median, frequencies, percentages and, crosstabs were calculated. Both bivariate and multivariate logistic regression analysis were done to identify associated factors of PH. Those variables having a p-value of < 0.25 in the bivariate analysis were entered to multivariable logistic regression. Those variables with a p value ≤ 0.05 were declared as significantly associated with PH. Finally, model fitness was checked by Hosmer and Lemshow test at p-value > 0.05 and it was 0.16. Results were organized and presented by using frequency tables and charts.

### Ethical consideration

Ethical clearance was obtained from Institutional Review Board (IRB) of College of Medicine and health science, University of Gondar and letter of permission was obtained from clinical director of UOGSCH before the actual data collection was started. Privacy and confidentiality of information was kept properly and names were not recorded.

## Result

### Socio-demographic and clinical characteristics

A total of 230 RHD patients were involved during the study period between September 2018 and September 2023. Most patients were young with median age of 28 years and interquartile range of 21 to 38 years. Among the study participants 72.2% were females and most of them were rural residents.

95% of the study participants experienced symptoms, with the most common ones being shortness of breath (90.4%), easy fatigability (87.3%), orthopnea (68.7%), and cough (61.7%). The majority of the patients (66.5%) had functional status in NYHA classes three and four. 40% of the participants in the study had symptoms for longer than three years, with a mean duration of symptoms of 45.6 months prior to the study period. The majority of participants (72.6%) had at least one heart failure-related hospitalization, while 13.5% had more than two hospitalizations. Ischemic stroke episodes diagnosed with brain CT scan were found in 11.7% of patients, and among those with ischemic stroke, most (70.4%) had atrial fibrillation (AF) and 59.3% had mitral stenosis (MS). AF was found in about 39% of RHD patients, of which 86.5% were taking anticoagulants. **(See** Table 1**)**

**Table 1:** Sociodemographic and clinical characterstis of RHD patients in adult echocardiography unit of university of Gondar hospital, Ethiopia. (*N* = 230)

Variable	Category	No PH (%)	PH (%)
**Age (Years)**	15–24	23(25.8)	66(74.2)
	25–34	19(27.9)	49(72.1)
	35–65	16(21.9)	57(78.1)
**Sex**	Male	15(23.4)	123(74.1)
	Female	43(25.9)	123(123)
Residence place	Urban	22(28.9)	54(71.1)
	Rural	36(23.4)	118(76.6)
**Presence of symptoms**	Symptomatic	48(21.9)	171(78.1)
	Asymptomatic	10(90.9)	1(9.1)
**NYHA functional class**	Class 1	12(80.0)	3(20.0)
	Class 2	22(35.5)	40(62)
	Class 3	9(11.7)	68(88.3)
	Class 4	15(19.7)	61(80.3)
**Symptom duration** **(months)**	≤ 36	38(27.5)	100(72.5)
	> 36	20(21.7)	72(78.3)
**Hospitalization frequency**	0	26(41.3)	37(58.7)
	1	15(16.3)	77(83.7)
	2	11(25.0)	33(75.0)
	≥ 3	6(19.4)	25(80.6)
**Ischemic stroke**	Yes	5(18.5)	22(81.5)
	No	53(26.1)	150(73.9)
**AF**	Yes	9(10.1)	80(89.9)
	No	43(30.5)	98(69.5)
IE	Yes	3(18.8)	13(81.3)
	No	55(25.7)	159(74.3)

### Echocardiographic variables and PH prevalence

**Fig. 1:**
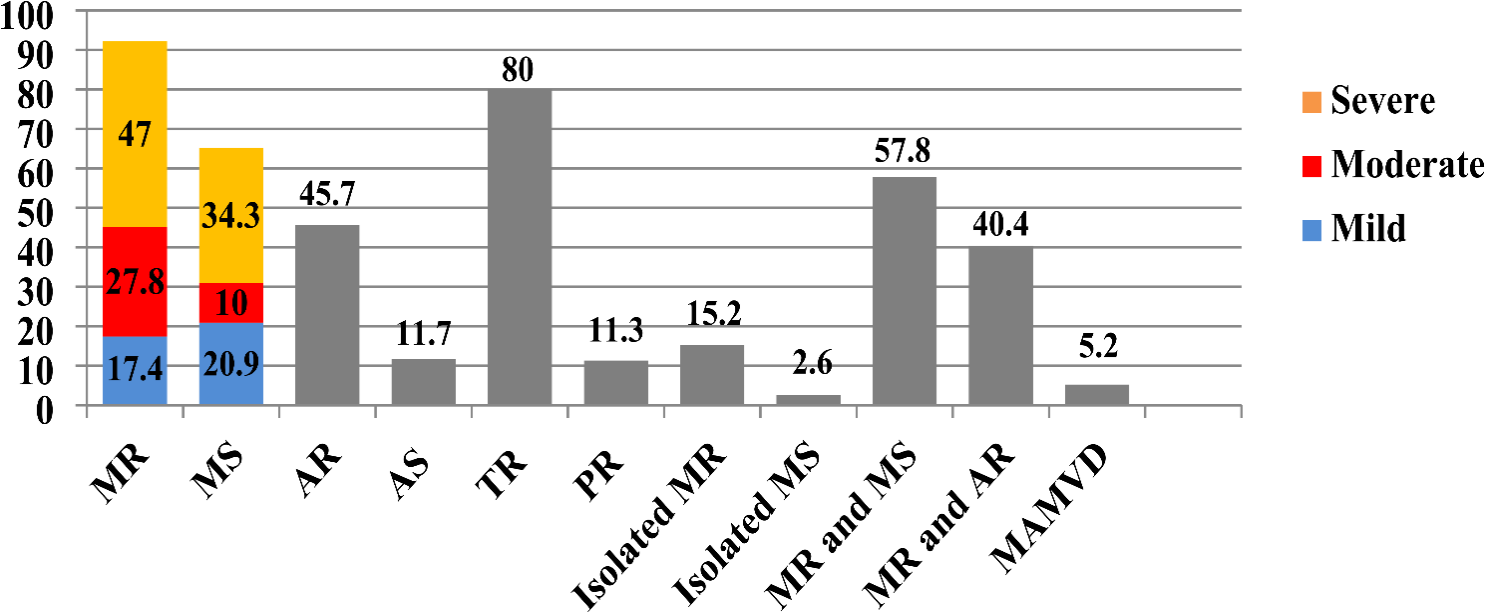
Patterns of Valvular abnormalities in RHD Patients in adult echocardiography Unit of University of Gondar hospital, Ethiopia.(*N* = 230)

The study found that the mitral valve was the most commonly affected valve in patients with RHD, followed by the aortic valve. In contrast, primary involvement of the tricuspid and pulmonary valves was uncommon, occurring in only 1.7% of cases. However, functional TR and PR without valvular thickening were observed in 80% and 11.3% of participants, respectively. MR was the most prevalent valvular abnormality, present in 91.7% of RHD patients. Furthermore, MS was found to coexist with MR in 57.8% of cases, while AR and MR coexisted in 40.4% of cases. Isolated MS (2.6%), mixed aortic and mitral valve disease (5.2%), and isolated aortic valve involvement (0.4%) were less common findings. (see Fig. 1).

Figure 1 Patterns of valvular involvement in patients with rheumatic heart disease (RHD). MR includes all patients with mitral regurgitation, while isolated MR refers to those with MR only, excluding other valve lesions. MS includes all patients with mitral stenosis, and isolated MS refers to those with MS alone, excluding other valve lesions. MAMVD represents mixed aortic (AR and/or AS) and mitral (MR and/or MS) valve disease. MR and MS denote patients with both MR and MS, and MR and AR includes those with regurgitant lesions affecting both the mitral and aortic valves. The severity of MR and MS is represented by blue, red, and yellow colors, while gray indicates different patterns of involvement without severity.

**Table 2:** Echocardiographic measurements and valvular lesions in RHD patients in adult echocardiography unit of university of Gondar hospital, Ethiopia.(*N* = 230)

Variables	Category	No PH (%)	PH (%)
**MR**	No	5(26.3)	14(73.7)
	Mild	21(53.8)	18(46.2)
	Moderate	13(20.3)	51(79.7)
	Severe	19(17.6)	89(82.4)
**MS**	No	5(26.3)	14(73.7)
	Non - Severe	21(53.8)	18(46.2)
	Severe	43(33.6)	85(66.4)
**AR**	No	34(27.2)	91(72.8)
	Mild	9(25.7)	26(74.3)
	Moderate	5(19.2)	21(80.8)
	Severe	10(22.7)	34(77.3)
**AS**	No	51(25.1)	152(74.9)
	Mild	3(33.3)	6(66.7)
	Moderate	4(36.4)	7(63.6)
	Severe	0(0)	7(100)
**TR**	No	38(82.6)	8(17.4)
	Mild	14(42.4)	19(57.6)
	Moderate	2(12.5)	14(87.5)
	Severe	4(3.0)	131(97.0)
**PR**	yes	3(11.5)	23(88.5)
	No	55(27.0)	149(73.0)
**LVEDD**	Normal	46(30.7)	104(69.3)
	Dilated	12(15.0)	68(85.0)
**LVESD**	Normal	46(29.7)	109(70.3)
	Dilated	12(16.0)	63(84.0)
**EF**	Preserved	51(26.4)	142(73.6)
	Reduced	7(18.9)	30(81.1)
**LA size**	Normal	32(68.1)	15(31.9)
	Dilated	26(14.2)	157(85.8)
**RA size**	Normal	51(72.9)	19(27.1)
	Dilated	7(4.4)	153(95.6)
**RV size**	Normal	52(68.4)	24(31.6)
	Dilated	6(3.9)	148(96.1)

In our study, we observed left ventricular end-diastolic diameter (LVEDD) dilatation in 34.8% of the patients, most of whom also had mitral regurgitation (MR) and left atrial (LA) dilatation. Right ventricular (RV) and right atrial (RA) dilatation were found in 67% and 69.6% of the patients, respectively, with most of them also having tricuspid regurgitation (TR). The majority of patients had preserved ejection fraction (EF) (> 50%), while mildly reduced and reduced EF were found in only 14% of patients with RHD (**see** Table 2).

**Fig. 2:**
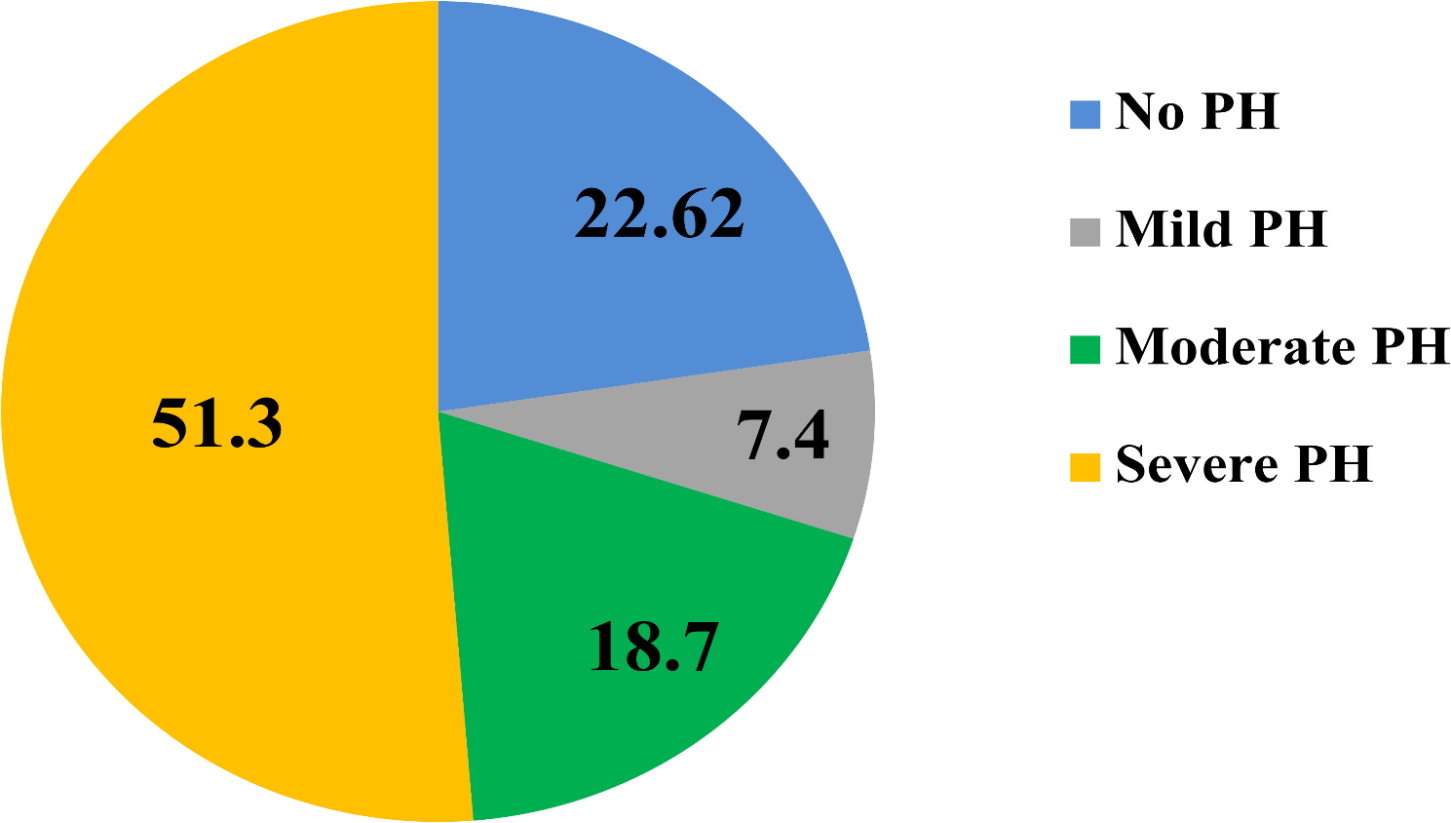
Pulmonary hypertension (PH) prevalence and severity among RHD patients in University of Gondar comprehensive specialized hospital, adult echocardiography unit

The prevalence of pulmonary hypertension (PH) was found to be high at 77.4% (95% CI: 71.4 − 82.6%) using echocardiographic estimated systolic pulmonary artery pressure (SPAP) > 35mmHg for diagnosis. Most patients (51.3%) had severe PH (≥ 60mmHg), while mild PH (36–44mmHg) and moderate PH (45-59mmHg) were found in 7.4% and 18.7% of patients, respectively. (see Fig. 2).

### Medications and surgical interventions

Only 9 participants (3.9%) underwent surgical interventions among which 6 had no PH post procedure. Majority of patients were taking diuretics (86.5%), whereas Beta-blocker, Digoxin and ACEI 43%, 23%, 10.9%. Warfarin was used as anticoagulant in 33.0%, while 4.3% were using aspirin. A total of 60.4% of participants were using rheumatic fever prophylaxis, among which Benzathine penicillin and Erythromycin were used in 33.5% and 27%, respectively (see Table 3).

**Table 3:** Medications used in RHD patients in adult echocardiography unit of university of Gondar hospital, Ethiopia. (*N* = 230)

Variable	Category	No PH (%)	PH (%)
**Diuretics**	Yes	37(18.6)	162(81.4)
	No	21(67.7)	10(32.3)
**Beta-blocker**	Yes	18(18.2)	81(81.8)
	No	40(30.5)	91(69.5)
**Digoxin**	Yes	3(5.7)	50(94.3)
	No	55(31.1)	122(68.9)
**ACEI**	Yes	4(16.0)	21(84.0)
	No	54(26.3)	151(73.7)
**Anticoagulation used**	Yes	13(15.1)	73(84.9)
	No	45(31.3)	99(68.8)
**Recurrence prophylaxis**	Yes	34(24.5)	105(75.5)
	No	24(26.4)	67(73.6)
**Surgical Intervention done**	Yes	6(66.7)	3(33.3)
	No	52(23.5)	169(76.5)

**Table 4:** Bivariate and multivariate analysis of associated factors of PH in RHD patients in adult echocardiography unit of university of Gondar hospital, Ethiopia(*N* = 230)

Variables	Category	No PH (%)	PH (%)	COR(95% CI)	*P* value	AOR(95% CI)	*P* value
**Age(year)**	15–24	23(25.8)	66(74.2)	1.00		1.00	
	25–34	16(23.5)	52(76.5)	1.13(0.54,2.36)	0.74	0.68(0.25,1.82)	0.44
	35–65	13(29.5)	31(70.5)	1.60(0.74,3.45)	0.22	1.002(0.33,2.97)	0.99
**Residence place**	Urban	21(27.6)	55(72.4)	1.00			
	Rural	31(20.1)	123(79.9)	1.51(0.80,2.86)	0.20	1.90(0.81,4.45)	0.13
**NYHA class**	1 and 2	32(41.6)	45(58.4)	1.00			
	3 and 4	20(13.1)	133(86.9)	4.72(2.46,9.08)	< 0.001	2.60(1.01,6.68)	0.04*
**Duration of symptoms categorized**	≤ 36 months	38(27.5)	100(72.5)	1.00			
	> 36 months	14(15.2)	78(84.8)	2.11(1.07,4.18)	0.031	1.73(0.71,4.22)	0.22
**Hospitalization frequency**	No admission	26(41.3)	37(58.7)	1.00			
	1	13(14.1)	79(85.9)	4.27(1.97,9.24)		1.23(0.43,3.51)	
	2	7(15.9)	37(84.1)	3.71(1.43,9.61)	0.007	0.61(0.15,2.37)	0.47
	≥ 3	6(19.4)	25(80.6)	2.93(1.05,8.14)	0.039	0.47(0.11,2.06)	0.32
**Moderate to severe MR**	Yes	24(41.4)	34(58.6)	3.63(1.87,7.03)	< 0.001	2.68(1.05,6.84)	0.03*
	No	28(16.3)	144(83.3)	1.00			
**Severe MS**	Yes	43(33.6)	85(66.4)	5.22(2.40,11.36)	< 0.001	5.31(1.87,15.06)	0.002*
	No	9(8.8)	93(91.2)	1.00			
**Mixed mitral valve disease**	Yes	24(18.0)	109(82.0)	1.84(0.98,3.43)	0.05	1.07(0.43,2.65)	0.87
	No	28(28.9)	69(71.1)	1.00			
**AF**	Yes	9(10.1)	80(89.9)	3.90(1.79,8.48)	0.001	2.64(0.86,8.11)	0.089
	No	43(30.5)	98(69.5)	1.00			
**Diuretics used**	Yes	32(16.1)	167(83.9)	9.48(4.14,21.70)	< 0.001	4.43(1.33,14.70)	0.015*
	No	20(64.5)	11(35.5)	1.00			
**BB use**	Yes	12(12.1)	87(87.9)	3.18(1.56,6.47)	0.034	1.46(0.53,4.03)	0.45
	No	40(30.5)	91(69.5)	1.00			

AOR = Adjusted odds ratio, AF = Atrial fibrillation, BB = beta blocker, CI = confidence interval, COR = crude odds ratio, MR = Mitral regurgitation, MS = Mitral stenosis, NYHA class = New York Heart association classification * significant association with p value < 0.05

### Factors associated with pulmonary hypertension

Binary logistic regression model was used to identify factors associated with PH. Variables with a p-value of < 0.25 on bivariate analysis were entered and analyzed by multivariate logistic regression to control the possible effects of confounders. 11 variables (shown below) had p value < 0.25 on bivariate analysis and were analyzed with multivariate logistic regression and those with p value of < 0.05 on multivariate analysis were taken as significant. Independent variables with high collinearity were identified and excluded one of them from analysis. This includes MS and LA and MR and LV dimensions.

Severe MS, moderate to severe MR, NYHA class 3 and 4, and Diuretics use were found to have significant association with p value < 0.05 on multivariate analysis (see Table 4). Patients having severe MS were found to have 5.3 times increased risk of having PH (AOR = 5.31,95% CI: 1.8,15.0) than those having mild and no MS and those with Moderate to severe MR had 2.6 times increased risk of developing PH(AOR = 2.68, 95%CI:1.05, 6.84). Diuretics use were associated with increased odds of having PH by 4.43 (95%CI: 1.33, 14.70) and advanced NYHA functional class (class 3 and 4) had increased the odds of PH by 2.60 (95%CI: 1.01, 6.68).

## Discussion

Rheumatic heart disease (RHD) causes significant morbidity and mortality in school-age children and young productive adults. This study has identified several complications, including pulmonary hypertension (77.4%), advanced NYHA class III and IV heart failure (66.5%), atrial fibrillation (38.7%), anemia (40%), stroke (11.7%), and valvular vegetations (7%). These complications were found to be higher in this study when compared with other studies in our literature review. This may be related to the shortage of surgical interventions done to RHD patients in our facility and, in general, in the country [15]. 

The prevalence of PH in this study was found to be significantly higher than most other studies, where prevalence ranges varied widely from 11 to 76.9%. Two multicenter studies - REMEDY study and a meta-analysis of PH prevalence and etiology in Africa - reported lower prevalence rates of 28% and 12.9%, respectively, using SPAP > 35mmHg for PH diagnosis. Similarly, two studies conducted in Uganda showed lower prevalence rates of 32.7% and 53.3% using SPAP > 30mmHg for PH diagnosis [2, 4, 8, 19]. On the other hand, relatively higher prevalence rates of PH were seen in studies conducted in Nigeria (72.1%), India (76.9%), and in MS patients in Iran (81%) [9, 14, 20]. The high prevalence of PH in our study could be due to the limited accessibility of surgical intervention in Ethiopia [15].

There were limited studies assessing the factors associated with PH. In our review, we found only one study that assessed the factors associated with PH in RHD patients. This study showed that moderate to severe mitral regurgitation (MR), severe mitral stenosis (MS), NYHA functional class III and IV, and diuretic use were significantly associated with PH.

The association between severity of mitral valve lesions and PH were also seen in other studies. This association is consistent with studies done in UK, USA, and Spain which showed that the degree of MR was associated with PH among patients with heart failure of different causes [12, 21, 22]. But we didn’t find a similar study in RHD patients. MR contributes to the development of pulmonary hypertension by elevating left atrial pressure, which is subsequently transmitted backward into the pulmonary vasculature, leading to increased pressure in the pulmonary arteries. Over time, sustained elevated pressure induces vasoconstriction and structural remodeling of the pulmonary vessels, a process characterized by thickening and stiffening of the vessel walls. This remodeling increases resistance within the pulmonary circulation, exacerbating the progression of PH. If left unmanaged, this chronic elevation in pressure and vascular damage perpetuates a cycle that significantly contributes to the worsening of pulmonary hypertension in individuals with MR [23].

The study revealed that severe MS was significantly associated with PH in RHD patients. A similar study from Iran on rheumatic MS patients also found a significant association between the severity of MS and PH [14]. This reinforces the notion that the hemodynamic changes associated with MS, particularly the increase in left atrial pressure, play a critical role in the onset of PH. In cases of severe MS, the narrowed mitral valve obstructs the flow of blood from the left atrium to the left ventricle, leading to a backlog of blood in the left atrium. This elevated atrial pressure is then transmitted to the pulmonary vasculature, causing increased pressure in the pulmonary arteries. The chronic elevation in pulmonary pressure triggers vascular remodeling, including the thickening and stiffening of the pulmonary vessels, which further increases resistance to blood flow and exacerbates PH. This process highlights the shared pathophysiological mechanisms between MR and MS in the development of pulmonary hypertension [23].

The study also found a significant association between advanced NYHA classes (III and IV) and pulmonary hypertension. This link can be explained by two mechanisms. First, Group 2 PH, which is often a consequence of left-sided heart failure, is associated with the progression of major adverse cardiac events. This leads to increased pulmonary venous pressure, which subsequently causes right heart strain and a decline in the NYHA functional class of patients. Second, both PH and advanced NYHA functional classes are manifestations of advanced cardiac disease. In this context, impaired left ventricular function leads to elevated pulmonary pressures, causing increased afterload on the right ventricle. Over time, this results in right ventricular dysfunction and further exacerbates the decline in functional status [6, 24].

In addition, the study found that the use of diuretics was associated with PH. Since PH can cause right-side heart failure and precipitate left-side HF, patients with PH are likely to be treated with diuretics [25]. A study conducted in Egypt on patients with HF also showed a similar association between PH and the use of diuretics [11].

These results indicate that PH is highly prevalent, which could increase morbidity and mortality. In order to lower PH and the morbidity that goes along with it, medical professionals are advised to focus on the early identification of severe valvular lesions in RHD patients and refer them to facilities that are prepared for surgical interventions. Expanding cardiac surgery facilities throughout Ethiopia is necessary, especially to serve school-age children, young adults, and rural communities. Prioritizing primary and secondary prevention strategies is essential to reducing complications related to RHD, as per the WHO’s 71st assembly agenda.

To improve outcomes for patients with pulmonary hypertension and heart failure, it is essential for health professionals to focus on the early detection of severe valvular lesions, and to refer them to facilities capable of providing surgical interventions. This would help reduce PH and the associated morbidity. For government policymakers, expanding cardiac surgery centers in Ethiopia to accommodate school-aged children, young adults, and rural populations is critical to addressing the growing burden of heart disease. Furthermore, as highlighted in the WHO 71st Assembly agenda, priority should be given to primary and secondary prevention strategies aimed at preventing complications from RHD. For researchers, RHD remains a neglected disease, and there are limited studies exploring its complications. This study, which assess factors associated with PH in Ethiopia, provides an important foundation for future research. Large-scale studies addressing RHD complications are needed to better understand the full scope of the disease and improve prevention and treatment strategies.

### Limitation of the study

One limitation of this study was that the echocardiographic method used to determine systolic PAP may not accurately reflect the presence of PH. The standard method for assessing PH is through a right heart catheterization (RHC). However, RHC is invasive and expensive, so most of the studies use echocardiography instead. Another limitation of this study is that it was conducted retrospectively, which limits some clinical and socio-demographic variables.

## Conclusion

The study found a high prevalence of pulmonary hypertension (77.4%) among patients with rheumatic heart disease. The presence of moderate to severe mitral regurgitation, severe mitral stenosis, NYHA class III and IV, and diuretic use were significantly associated with pulmonary hypertension.

## Electronic supplementary material

Below is the link to the electronic supplementary material.

Supplementary Material 1

## Data Availability

All pertinent data are comprised in the manuscript. The dataset is available and can be obtained from the corresponding author upon reasonable request.
