# Supplementary material for: Prevalence and determinants of pulmonary hypertension in rheumatic heart disease patients at university of Gondar comprehensive specialized hospital: a retrospective study from 2018 to 2023
Source: BMC Cardiovasc Disord. 2025 Mar 18;25:193. doi: 10.1186/s12872-025-04648-1 (PMC11916979; doi:10.1186/s12872-025-04648-1)
Supplement: Supplementary file 1 — Supplementary Material 1 [file 12872_2025_4648_MOESM1_ESM.pdf]

Questionnaires

Questionnaires on prevalence and predictors of Pulmonary hypertension in RHD patients at University of Gondar Hospital echocardiography unit

Date of data collection_____________

Medical registration number ___________

Part 1: Socio-demographic characteristics and habits

SN	Variables	
1.1	Age	_____years
1.2	Sex	1. Male<br>2. Female
1.3	Residence	1. Urban<br>2. Rural

Part 2: clinical characteristics and comorbidity

SN	Variables	
2.1	NYHA Class	1. I<br>2. II<br>3. III<br>4. Iv
2.2	Duration of symptoms	________ in months
2.3	Symptoms (shortness of breath , orthopnea, PND, palpitation, leg swelling, and fatigue)	1. Yes<br>2. No
2.4	BP	_______mmHg
2.5	Hospital admission frequency	1. Once a year<br>2. twice a year<br>3. >3 times /year
2.7	Stroke	1. Yes

		2. No
2.8	Hg	_____mg/dl
2.9	PITC	1. Non-Reactive<br>2. Reactive<br>3. Unknown status

Part 3: Echocardiography and ECG parameters

SN	Variables		Mild	moderate	severe
3.1	Valvular lesion type and severity	1. MR			
		2. MS			
		3. AR			
		4. AS			
		5. TR			
		6. PR			
3.2	LVEDD	______mm			
3.3	LVESD	______mm			
3.4	EF	______%			
3.5	LA	______mm			
3.6	RA	______mm			
3.7	RV	______mm			
3.8	LV	______mm			
3.9	Systolic PAP	______mmHg			
3.10	AF	1. Yes<br>2. No			
3.11	RBBB	1. Yes<br>2. No			
3.12	RVH	1. Yes<br>2. No			

Part 4: medications and intervention

SN	Variables	
4.1	Diuretics type and dose	1. ________<br>2. ________<br>3. ________
4.2	ß-blocker	1. _________<br>2. _________
4.3	Digoxine	1. Yes<br>2. No
4.4	ACEI	1. yes<br>2. No
4.5	Anticoagulation	_________
4.6	Secondary prophylaxis	_________
4.7	Surgical intervention	_________
